# Defects in *PDIA4* increase individuals’ susceptibility to congenital heart disease

**DOI:** 10.3389/fgene.2026.1753969

**Published:** 2026-03-04

**Authors:** Yuquan Lu, Jiangjie Liu, Siyu Sun, Zhiyu Feng, Yuan Gao, Shaojie Min, Quannan Zhuang, Siyi Lin, Quming Zhao, Xianghui Huang, Wei Sheng, Guoying Huang

**Affiliations:** 1 Pediatric Heart Center, Children’s Hospital of Fudan University, Shanghai, China; 2 Shanghai Key Laboratory of Birth Defects, Children’s Hospital of Fudan University, Shanghai, China; 3 Fujian Key Laboratory of Neonatal Diseases, Xiamen Children’s Hospital, Xiamen, Fujian, China

**Keywords:** congenital heart disease, protein disulfide isomerase family member 4, targeted sequencing, variants, whole-exome sequencing, wnt/β-catenin signaling pathway

## Abstract

**Introduction:**

Congenital heart disease (CHD) comprises structural abnormalities of the heart and major blood vessels arising during fetal development. Protein disulfide isomerase family member 4 (PDIA4) facilitates protein folding processes. However, its potential involvement in CHD has not been investigated. In this study, we identified *PDIA4* as a candidate gene potentially involved in cardiac development.

**Methods:**

Whole-exome sequencing and targeted sequencing were performed to identify *PDIA4* as a candidate gene of CHD. To investigate the functional role of *PDIA4*, *PDIA4*-knockdown human umbilical vein endothelial cells were generated, followed by cellular and transcriptomic analyses.

**Results:**

A *de novo PDIA4* mutation (NM004911: c.1249G>A: p.V417I) was found in a patient with complex CHD. Burden analysis demonstrated a significant enrichment of rare deleterious *PDIA4* variants in patients with CHD compared with controls (Person’s chi-squared test: OR: 4.08, 95% CI: 2.23–4.76, *p* = 7.46e−7). Deficiency of *PDIA4* in human umbilical vein endothelial cells suppressed functionality and inhibited the protein levels of both total and nuclear β-catenin as well as the downstream activity of the WNT/β-catenin signaling pathway.

**Conclusion:**

Our study suggests that *PDIA4* may act as a susceptibility gene for CHD, and its deficiency may contribute to abnormal cardiac development by modulating the WNT/β-catenin signaling pathway.

## 1 Introduction

Congenital heart disease (CHD) refers to cardiovascular malformations that occur during the fetal period. Severe CHD can cause substantial clinical challenges, affecting patients’ long-term health (Sprong et al., 2021). Consequently, elucidating the genetic etiology and molecular mechanisms underlying CHD is of great importance for early prevention, diagnosis, and treatment (Pierpont et al., 2007). Extensive studies have explored the genetic basis of CHD and identified pathogenic variants in multiple genes, including NOTCH1, HEY2, TRAF7, and WDR62 (Stanley et al., 2024; Van Walree et al., 2021; Mishra-Gorur et al., 2023; Hao et al., 2022). In addition, variants in other gene have been confirmed to be closely associated with multiple CHD subtypes, including ventricular septal defect, atrial septal defect, tetralogy of Fallot, and patent ductus arteriosus, by regulating gene expression to affect cardiac structure development (Zuo et al., 2024a; Zuo et al., 2024b; Zuo et al., 2023; Zuo et al., 2022).

Cardiomyocytes and endocardial cells play critical roles in heart development and are the principal cell types implicated in CHD (Tambi et al., 2023). Dysfunction of endocardial cells can lead to reduced cardiac chamber volume and abnormal ventricular cavity formation (Miao et al., 2020; Serrano et al., 2019; Liu et al., 2023). Human umbilical vein endothelial cells (HUVECs) serve as a representative model for cardiogenesis and cardiovascular pathogenesis because of their accessibility and functional similarities to endocardial cells (Liang et al., 2021; La Rocca et al., 2009).

Advances in sequencing technology have improved the detection of CHD-associated genetic variants. Whole-exome sequencing (WES) has become an effective approach for identifying pathogenic variants, including deleterious missense, loss-of-function, and structural variants. These studies provide a molecular foundation for elucidating the pathogenic mechanism underlying sporadic CHD in offspring of clinically unaffected parents, indicating *de novo* mutations as potential contributors to the disease (Pierpont et al., 2007; Homsy et al., 2015; Sifrim et al., 2016).

Protein disulfide isomerase family member 4 (PDIA4) is a member of the protein disulfide isomerase (PDI) family. It is involved in protein folding within the endoplasmic reticulum. Previous studies have reported that some PDI family members are related to the development of the heart. For example, loss of PDIA6 in zebrafish results in pericardial edema and abnormal lateralization during early embryogenesis (Hoshijima et al., 2002). Cardiac defects have also been observed in *PDIA*10-knockdown zebrafish (Wang et al., 2014). Aberrant protein expression of PDIA4 has been proven to participate in the pathogenesis of cancer and diabetes (Kang et al., 2023; Kuo et al., 2021). Notably, data from the UCSC Genome Browser indicate elevated PDIA4 expression in endocardial cells of the developing mouse heart (Perez et al., 2025). Meanwhile, single-cell profiling data from the Human Developmental Cell Atlas (HDCA) reveal high PDIA4 expression in endocardial cells within the human embryonic heart (Haniffa et al., 2021). However, the association between *PDIA4* and CHD, as well as its regulatory mechanism, remains unclear.

In this study, we conducted WES on a family with complex CHD and identified a *de novo PDIA4* mutation. Targeted sequencing of 1,792 sporadic patients revealed an association between rare deleterious PDIA4 variants and CHD occurrence (Person’s chi-squared test: OR: 4.08, 95% CI: 2.23–4.76, *p* = 7.46e-7). Functional analyses revealed that PDIA4 deficiency disrupts endothelial cell function and is associated with attenuated WNT/β-catenin signaling. Collectively, these findings reveal that *PDIA4* may be a novel susceptibility gene potentially contributing to abnormal cardiac development.

## 2 Materials and methods

### 2.1 Study subjects

This study enrolled a pedigree with complex CHD and 1,792 patients with sporadic CHD at the Children’s Hospital of Fudan University. All procedures were conducted in alignment with the principles of the Declaration of Helsinki (2013 revision). Written informed consent was obtained from the parents or legal guardians of all participating patients. The study protocol received approval from the Ethics Committee of the Children’s Hospital of Fudan University [No. (2021)429].

### 2.2 Whole-exome sequencing and targeted sequencing

Genomic DNA was extracted from peripheral blood samples of the patients and their parents using the QIAamp DNA Blood Mini Kit (QIAGEN, Germany). WES was performed on the proband and her parents by Gemple Biotech Co., Ltd. (Shanghai, China). Targeted sequencing of PDIA4 was conducted on cases of sporadic CHD. The criteria for screening rare and deleterious variants were as follows: (1) loss-of-function variants; (2) missense mutations with absence or minor allele frequency ≤ 0.01% in gnomADv2_exome_EAS and a combined annotation dependent depletion (CADD) score >20. Allele frequencies from healthy controls were obtained from East Asians in the Genome Aggregation Database Version 2 (gnomADv2), accessible at https://gnomad.broadinstitute.org/. The same filtering criteria were applied to both patient and control datasets to ensure consistency.

Reference control data were sourced from GnomADv2, which includes 125,748 exome sequences. Considering ethnic specificity, we restricted our analysis to East Asian individuals (*n =* 9,197) from gnomADv2 as the control group. The filtering criteria for rare deleterious variants in the control population were consistent with those applied to our CHD patients.

### 2.3 Cell culture

HEK293T cells were cultured in Dulbecco’s modified Eagle’s medium (1×) (Gibco, United States) containing 10% fetal bovine serum (Excell, Uruguay). HUVECs were maintained in endothelial cell medium (ScienCell, United States). All cells were incubated at 37 °C and 5% CO_2_. For passaging, cells were washed with phosphate-buffered saline (PBS) (Biosharp, China) and then detached using 0.25% trypsin-EDTA (Gibco, United States)

### 2.4 Generation of PDIA4-knockdown HUVEC cell lines

Short hairpin RNA (shRNA) sequences targeting PDIA4 were designed and synthesized by Genomeditech Company and subsequently inserted into PGMLV lentiviral vectors (Supplementary Table S1). The constructed lentiviral vector contained a puromycin resistance gene for the selection of stable PDIA4-knockdown cell lines. HEK293T cells were seeded into 10-cm dishes and grown to reach 80% confluence. They were then transfected with a mixture of the constructed PGMLV-PDIA4-shRNA plasmid (6.8 μg) and the lentiviral packaging plasmids PMDL2 (3.4 μg) and PSPAX (5.2 μg), using Lipofectamine 3000 transfection reagent following the manufacturer’s guidelines. Viral supernatants were collected 48 h after transfection and concentrated according to standard protocols. HUVECs were seeded in 6-well plates and infected with the concentrated lentiviral supernatant when they reached approximately 90% confluence. After 72 h, infected cells were selected with puromycin (Beyotime, China) for 2 weeks. Knockdown efficiency was ultimately confirmed by Western blot analysis.

### 2.5 Reverse transcription–quantitative real-time polymerase chain reaction

Total RNA was extracted using TRIzol reagent (Thermo Fisher Scientific, United States). RNA was reverse-transcribed into complementary DNA (cDNA) using the PrimerScript™ RT Reagent Kit (Takara, Japan). Quantitative polymerase chain reaction (PCR) (qPCR) was performed using TB Green® Premix Ex Taq™ (TaKaRa, Japan) on a QuantStudio Real-Time PCR System (Thermo Fisher Scientific, United States), according to the manufacturer’s guidelines. Primer sequences are listed in Supplementary Table S1.

### 2.6 Western blot

HUVECs were lysed in protein lysate (Beyotime, China) containing 50× protease inhibitor (Beyotime, China), 50× salubrinal (Beyotime, China), and 50× EDTA (Beyotime, China). Lysates were centrifuged, and protein concentrations were measured using a BCA Protein Assay Kit (TaKaRa, Japan). Protein samples, boiled after mixing with 5 x loading buffer, were separated by 7.5% SDS-polyacrylamide gel (Epizyme, China) and subsequently transferred to a PVDF membrane (Millipore, United States). The membrane was blocked with 5% non-fat milk at ambient temperature for 2 h and then incubated at 4 °C overnight with primary antibodies against PDIA4 (Proteintech, 1D5F3) at 1:5000, β-catenin (CST, 8480) at 1:1000, lamina/c (CST, 2032) at 1:1000, and glyceraldehyde-3-phosphate dehydrogenase (GAPDH) (CST, 2118) at 1:1000. After six washes with TBST (Beyotime, ST673), it was incubated with secondary antibodies (CST, 7074/7076) diluted 1:2000 for 2 h at room temperature. Finally, protein bands were visualized with the ChemiDoc Imaging System (Bio-Rad XRS+).

### 2.7 Cell proliferation assay

Cell proliferation was assessed using the Cell Counting Kit-8 (CCK8, Dojindo, Japan). HUVECs were seeded at a density of 8000 cells per well in 96-well plates. CCK8 solution (10 µl) was added to each well at 0 h, 24 h, 48 h, and 72 h. Absorbance at 450 nm was measured after incubation for 2 h.

### 2.8 5-ethynyl-2′-deoxyuridine assay

Cell proliferation was assessed using the BeyoClick 5-ethynyl-2′-deoxyuridine (EdU)-555 Cell Proliferation Detection Kit (Beyotime, China). HUVECs were seeded in 6-well culture plates and cultured to approximately 80% confluence. The EdU working buffer was added to each well, followed by a 2-h incubation. Fluorescently labeled cells were imaged using a fluorescence microscope. ImageJ software was utilized to analyze the final data.

### 2.9 Wound-healing assay

HUVECs were seeded into 6-well culture dishes and cultured to full confluence. A 10 µl pipette tip was used to form a wound. Floating cells were removed by washing twice with PBS, and cells were then incubated in serum-free medium. Cell migration dynamics were documented and quantified by measuring the residual wound area from microscopic images captured at 0 h and 8 h with ImageJ software.

### 2.10 RNA sequencing

Total RNA was extracted from three independent biological replicates with TRIzol reagent (Invitrogen, United States), following the manufacturer’s instructions. The subsequent reverse transcription and high-throughput sequencing procedures were performed by Novogene Company (Shanghai, China), following standard service protocols.

### 2.11 Nuclear and cytoplasmic fractionation assays

Cells were harvested using the same procedure used for passaging and collected as a cell pellet. Cytoplasmic protein extraction reagent A (200 μL; Beyotime, P0028) was added to each 20 μL cell pellet, followed by vortexing for 5 s and incubation on ice for 10 min. Cytoplasmic protein extraction reagent B (10 μL; Beyotime, P0028) was then added, and the samples were vortexed for an additional 5 s and incubated on ice for 1 min. The lysates were centrifuged at 12,000 × g at 4 °C for 5 min, and the supernatants containing cytoplasmic proteins were collected.

The remaining pellet was resuspended in 50 μL of nuclear protein extraction reagent (Beyotime, P0028), vortexed vigorously for 15–30 s, and incubated on ice for 30 min, with intermittent vortexing every 1–2 min. Following centrifugation at 12,000 × g at 4 °C for 10 min, the supernatants containing nuclear proteins were collected for subsequent analysis.

### 2.12 Statistical analysis

All data are presented as the mean ± standard deviation (SD) from three independent experiments. Statistical analyses were performed using GraphPad Prism 9.5. Student’s t-test and Pearson’s chi-squared test were applied as appropriate. *p* < 0.05 was considered statistically significant. Figures were generated using GraphPad Prism 9.5.

## 3 Results

### 3.1 Identification of a *PDIA4 de novo* mutation in a CHD pedigree

**FIGURE 1:**
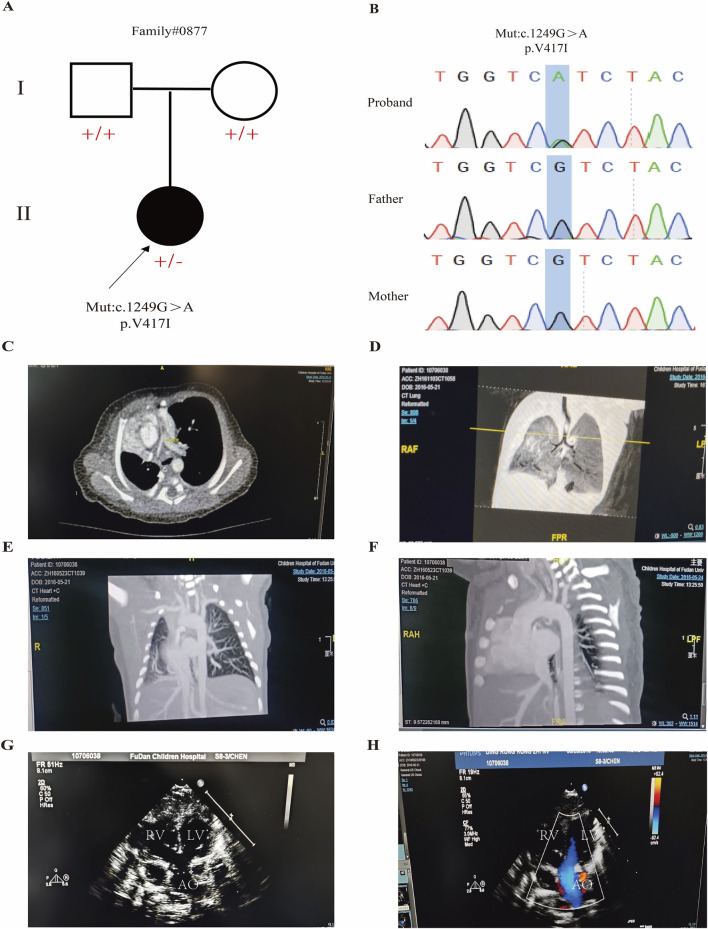
*de novo* mutation in *PDIA4* identified in a complex CHD family. **(A)** Pedigree of a family with complex CHD, showing the proband carrying a *de novo* mutation in the *PDIA4* gene (c. 1249 G>A). **(B)** Sanger sequencing of the proband and her parents. **(C–F)** Chest computed tomography (CT) for CH0877 carrying a *de novo* mutation (*PDIA4*: c. 1249 G>A: p. V417I), indicating congenital pulmonary dysplasia. **(G,H)** Echocardiography image for CH0877 carrying a *de novo* mutation indicating TOF, VSD, and ASD. TOF, tetralogy of Fallot; VSD, ventricular septal defect; ASD, atrial septal defect; PAPVR, partial anomalous pulmonary venous return.

To investigate potential genetic variants in CHD, we performed WES on an infant with complex CHD, including tetralogy of Fallot (TOF), mesocardia, ventricular septal defect (VSD), atrial septal defect (ASD), partial anomalous pulmonary venous return (PAPVR) of the right lung, and congenital pulmonary dysplasia, along with her unaffected parents (Figures 1C–H). We found that the proband carried a *de novo* mutation (NM_004911: c.1249G>A; p.V417I) in *PDIA4* (OMIM: 620018), which was absent in the gnomADv2 database, indicating it is rare (Figure 1A). *In silico* pathogenicity prediction using multiple bioinformatics tools suggested that this mutation is potentially deleterious. MutationTaster classified it as “disease causing,” while PolyPhen-2 predicted it to be “probably damaging” with a confidence score of 0.994, and the combined annotation-dependent depletion (CADD) score was 20.7 (Table 1). Then, we collected peripheral venous blood from the family members and confirmed the *de novo* mutation by Sanger sequencing (Figure 1B).

**TABLE 1:** Pathogenicity prediction of the de novo variant in the PDIA4 gene.

No. of the patient (family)	Position	Variant	Protein change	GnomAD_exome_EAS	MutationTaster	PolyPhen2	CADD_phred score	Pattern of inheritance
No_0877	ch7:148703028	c.1249G>A	p.Val417Ile	NA	D	D	20.7	De novo

NA, not available, indicating a missing database value; D, disease-causing, indicating pathogenicity.

### 3.2 Evolutionary conservation and structural characterization of the *de novo PDIA4* mutation

**FIGURE 2:**
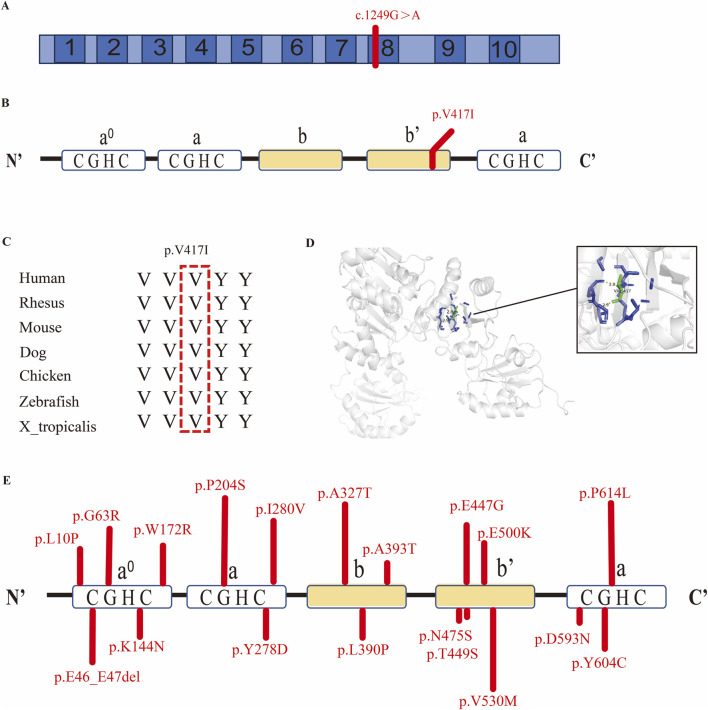
Analysis of the rare pathogenic *PDIA4* variants. **(A)** Location of the *de novo* variant in the *PDIA4* gene. **(B)** Location of the p.V417I amino acid variant in the PDIA4 protein. **(C)** Conservative analysis of the amino acid p.V417I and its surrounding sequence across multiple species. **(D)** Predicted three-dimensional structure of the PDIA4 protein. **(E)** Distribution of rare pathogenic variants in the PDIA4 protein.

The identified *de novo* mutation is located in exon 8 of the *PDIA4* gene, resulting in the substitution of valine with isoleucine at amino acid position 417, which is primarily located within the b' domain of PDIA4 (Figures 2A,B), a non-catalytic domain potentially involved in substrate binding (Freedman et al., 2002). Evolutionary conservation analysis via the UCSC Genome Browser revealed that the amino acid at position 417 is highly conserved across diverse species, indicating its functional importance (Figure 2C). Structural modeling of PDIA4 was generated by SWISS-MODEL. Subsequent analysis in PyMOL revealed an inter-residue distance of 2.9 Å (<5 Å) between valine 417 and its contacting residues, indicating potential interactions between these residues. (Figure 2D).

### 3.3 Rare deleterious *PDIA4* variants are associated with sporadic CHD

To further evaluate the association between PDIA4 and CHD, we performed targeted sequencing of *PDIA4* in a cohort of 1792 sporadic CHD patients. The clinical profile summary of the cohort is summarized in Supplementary Table S2, with patients classified into seven CHD subtypes (Geng et al., 2014). The results identified 19 rare deleterious *PDIA4* variants that fulfilled the filtering criteria (a. loss of function; b. missense mutations: absence or minor allele frequency ≤0.01% in gnomADv2_exome_EAS, and CADD score >20) (Table 2). These variants were distributed across distinct functional domains of the PDIA4 protein. (Figure 2E). Applying identical filtering criteria, we identified 24 rare deleterious variants from the control group in the gnomADv2_exome_EAS database (Supplementary Table S3). Comparison of variant frequencies revealed a significantly higher burden of rare deleterious *PDIA4* variants in CHD patients (19/3,584, 0.53%) than in controls (24/18,394, 0.13%). We found that these variants were associated with the occurrence of CHD (Person’s chi-squared test: OR: 4.08, 95% CI: 2.23–4.76, p = 7.46e−7) in Table 3. As shown in Supplementary Table S4, these variants were predominantly enriched in conotruncal defect (CTD) patients. We stratified the study cohort into a CTD group and a non-CTD group. The results demonstrated that the frequency of rare deleterious *PDIA4* variants in the CTD group (14/1,386, 1.01%) was higher than that in the non-CTD group (5/1,672, 0.2) in Supplementary Table S5 (Pearson’s chi-squared test: OR: 3.40; 95% CI: 1.22–9.46; *p* = 0.013).

**TABLE 2:** Identification of 19 rare deleterious variants in sporadic CHD patients.

Patient ID	HGVS	Annotation	ge_EAS	SIFT	PolyPhen2	MutationTaster	CADD
951	c.1340A>G p.E447G	Missense	-	D	D	D	32
3759	c.1777G>Ap.D593N	Missense	0	T	B	D	22.7
1931	c.514T>C	Missense	-	D	D	D	28.6
	p.W172R						
982	c.29T>C	Missense	-	D	B	N	18.49
	p.L10P						
2207	c.1841C>Tp.P614L	Missense	5.81E-05	D	D	D	31
2501, B1173	c.1811A>Gp.Y604C	Missense	0	D	D	D	29.8
193	c.1588G>Ap.V530M	Missense	0	D	D	D	24.8
99	c.1498G>Ap.E500K	Missense	0	T	B	D	24.2
247	c.1424A>Gp.N475S	Missense	0	T	D	D	25.6
B849	c.1346C>Gp.T449S	Missense	-	T	B	D	22.6
1346, B1038	c.1177G>Ap.A393T	Missense	-	T	B	N	12.48
B495	c.1169T>C	Missense	0	T	B	D	20.8
	p.L390P						
1154	c.979G>A	Missense	6.15E-05	D	D	D	29.7
	p.A327T						
S28	c.832T>G	Missense	5.89E-05	D	D	D	27.2
	p.Y278D						
B108	c.187G>A	Missense	-	D	D	D	27.9
	p.G63R						
2182	c.610C>T	Missense	-	D	D	D	24.7
	p.P204S						
1143	c.432G>T	Missense	-	T	D	D	29.5
	p.K144N						
0243	c.838A>G	Missense	-	T	B	N	10.72
	p.Ile280Val						
B120	c.135_140delGGAGGA p.E46_E47del	Disruptive	-	-	-	-	31
		inframe					
		deletion					

**TABLE 3:** Genetic findings in congenital heart defect patients with PDIA4 variants.

Comparison of rare deleterious PDIA4 variants in sporadic CHD and control group	Allele count (identified PDIA4 variants)	Allele count (no identified PDIA4 variants)	*p*-value	OR	95% CI lower	95% CI upper
CHD population	19	3565	7.46e−7	4.08	2.23	4.76
Database (GnomAD v2_EAS)	24	18370				

Person’s chi-squared test; OR, odds ratio; CI, confidence interval; GnomADv2_EAS, East Asians in Genome Aggregation Database Version 2.

### 3.4 PDIA4 deficiency impairs the proliferation and migration of HUVECs

**FIGURE 3:**
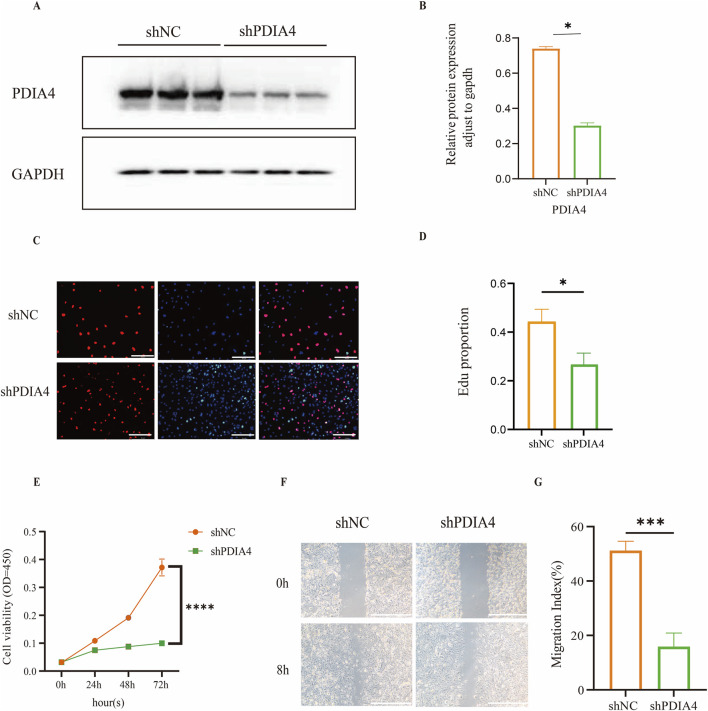
PDIA4 deficiency attenuates the function of HUVECs. **(A)** Western blot analysis of PDIA4 protein expression in the shNC and shPDIA4 groups. **(B)** Quantification of PDIA4 protein levels normalized to GAPDH. n = 3, *p < 0.05. **(C)** Representative EdU staining of proliferating cells. Scale bars, 200 µm. **(D)** Quantification of the EdU^+^/DAPI^+^ cell ratio between the shNC and shPDIA4 groups. n = 3, *p < 0.05. **(E)** Analysis of proliferation using the CCK-8 assay. n = 3, ****p < 0.0001 using the Student’s t-test. **(F)** Representative images of the wound-healing experiment for migration. Scale bars, 200 µm. **(G)** Quantification of the migration index between the shNC and shPDIA4 groups. n = 3, ***p < 0.001.

To explore the functional role of PDIA4 in HUVECs, we generated PDIA4-knockdown (shPDIA4) HUVECs using shRNA-mediated lentiviral transduction. Western blot analysis confirmed a marked reduction in PDIA4 protein expression in the shPDIA4 group compared with the scrambled shRNA-transduced negative control (shNC) (Figures 3A,B). Cell proliferation was evaluated using EdU and CCK-8 assays. The EdU assay revealed a significant decrease in the proportion of proliferating cells following PDIA4 knockdown (Figures 3C,D). Consistently, the CCK-8 assay indicated significantly reduced cell proliferation in the shPDIA4 group compared with the shNC group at 72 h (Figure 3E). A wound-healing assay was performed to assess the effect of PDIA4 deficiency on cell migration. Quantitative analysis indicated that PDIA4 deficiency significantly attenuated the migratory capacity of HUVECs at 8 h (Figures 3F,G).

### 3.5 PDIA4 deficiency suppresses WNT/β-catenin signaling in HUVECs

**FIGURE 4:**
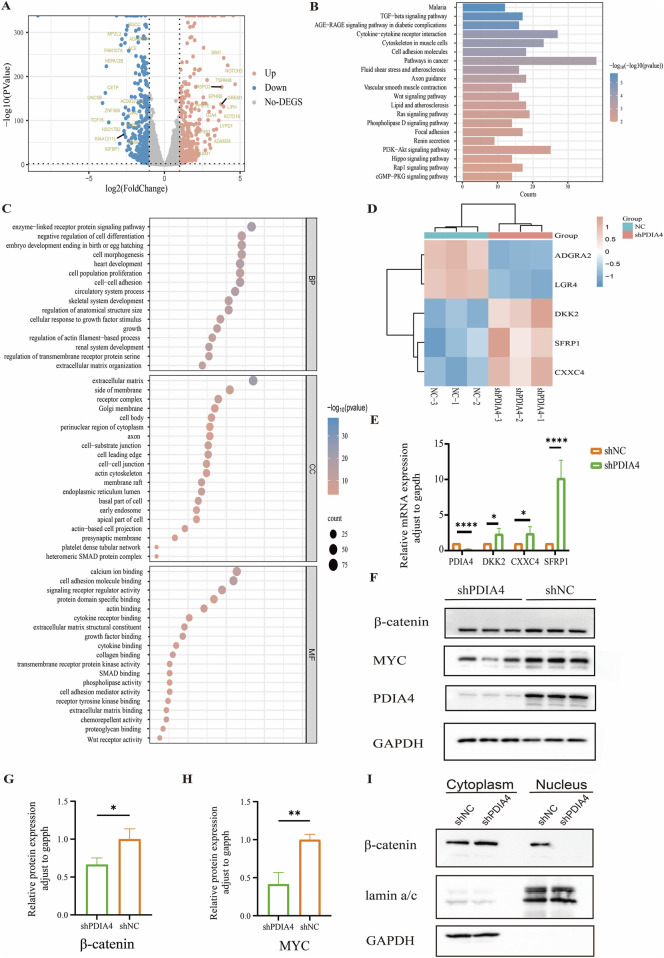
PDIA4 deficiency is associated with suppression of WNT/β-catenin signaling. **(A)** Volcano plot showing 3,875 upregulated genes (red dots) and 3,955 downregulated genes (blue dots). **(B)** KEGG pathway analysis of differentially expressed genes. **(C)** GO analysis of differentially expressed genes in BP, CC, and MF terms. **(D)** Heatmap of differentially expressed genes related to the WNT signaling pathway. n = 3. **(E)** Validation of the expression levels of differentially expressed genes associated with the WNT signaling pathway using RT-qPCR. *p < 0.05, ****p < 0.0001. **(F)** Western blot analysis of total β-catenin and MYC protein levels. **(G)** Quantification of β-catenin protein expression normalized to GAPDH. n = 3, *p < 0.05. **(H)** Quantification of MYC protein expression normalized to GAPDH. n = 3, **p < 0.01. **(I)** Western blot analysis of nuclear and cytoplasmic β-catenin protein levels.

Transcription profiling and functional enrichment analyses were performed in the shPDIA4 and shNC groups to explore the molecular mechanism by which PDIA4 modulates HUVEC function. A total of 3,875 genes were notably upregulated and 3,955 were markedly downregulated in the shPDIA4 group compared with the shNC group, as visualized in the volcano plot (Figure 4A). Kyoto Encyclopedia of Genes Genome (KEGG) pathway analysis of the differentially expressed genes revealed significant enrichment of pathways related to cardiac development, including the WNT signaling pathway and cytoskeleton in muscle cells (Figure 4B). Gene Ontology (GO) enrichment analysis identified biological processes related to cell proliferation and heart development (Figure 4C).

As shown in Figure 4D, a heatmap highlighted the upregulation of multiple inhibitors of the canonical WNT/β-catenin signaling pathway, including *DKK2*, *CXXC4*, and *SFRP1* (Shih et al., 2007; Filleur et al., 2009; Hirata et al., 2009). RT-qPCR validation confirmed a significantly increased expression of these genes in the shPDIA4 group compared with the shNC group (Figure 4E). Given the central role of β-catenin in canonical WNT signaling, the protein level of β-catenin and its downstream effector MYC, which is associated with cell proliferation, were further examined (Wang et al., 2023a; Qu et al., 2018). Western blotting demonstrated that total cellular levels of β-catenin and MYC were downregulated in the shPDIA4 group (Figures 4F–H). In addition, the main activated sign of the canonical WNT/β-catenin signaling pathway is the accumulation of β-catenin in the nucleus (Logan and Nusse, 2004). Nuclear and cytoplasmic fractionation assays revealed impaired β-catenin nuclear translocation in the shPDIA4 group (Figure 4I), indicating suppression of WNT/β-catenin signaling activity.

## 4 Discussion

CHD ranks among the most common congenital anomalies in newborns, with an incidence of approximately 6‰–10‰ among live births (Triedman and Newburger, 2016; Van Der Linde et al., 2011). Accumulating evidence indicates that genetic factors are associated with severe and sporadic forms of CHD (Acuna-Hidalgo et al., 2016). Here, we provided the first genetic and functional evidence implicating PDIA4 as a potential susceptibility gene for CHD.

WES effectively identifies *de novo* mutations among patients with CHD. Likewise, it allows for the identification of pathogenic mutations in genes that are vital to cardiac development within sporadic cases of CHD (Zaidi et al., 2013; Jin et al., 2017). In this study, an investigation was conducted on a child with complex CHD. WES of the family trio identified a *de novo* mutation (c.1249G>A) in the *PDIA4* gene. We performed Sanger sequencing on the proband and her unaffected parents, confirming that the identified mutation was *de novo*. Then we performed targeted sequencing of the *PDIA4* in a large cohort of sporadic CHD patients. Compared with the control group, the rare deleterious *PDIA4* variants were associated with the occurrence of CHD. Subgroup analysis demonstrated that these variants were particularly enriched in patients with CTD.

Researchers have increasingly recognized the critical roles of endothelial cells, one of which regulates cardiac development (D’Amato et al., 2016; Rhee et al., 2018). In our study, we revealed that PDIA4 deficiency reduced HUVEC proliferation and compromised their migratory capacity. This result was in line with previous reports in which endothelial cell-specific PDIA4 depletion leads to impaired autophagic flux, accompanied by endothelial dysfunction and apoptosis (Bu et al., 2024). These analyses suggest that PDIA4 is important for maintaining normal endothelial cell behavior.

Transcriptomic and proteomic analyses were performed to explore the mechanisms underlying HUVEC dysfunction induced by PDIA4 deficiency. Our data suggest that PDIA4 deficiency is associated with suppression of canonical WNT/β-catenin signaling, as reflected by upregulation of inhibitors of the WNT signaling pathway, reduced total protein levels of β-catenin and MYC, and impaired nuclear translocation of β-catenin. The WNT/β-catenin signaling pathway regulates cell proliferation, migration, and differentiation (Wang et al., 2023b; Ueno et al., 2007). β-catenin acts as a co-activator of LEF/TCF family transcription factors, facilitating the transcription of downstream target genes (Logan and Nusse, 2004). In this context, attenuation of this pathway provides a plausible mechanistic explanation for the functional deficits observed in PDIA4-deficient HUVECs.

In summary, our findings provide novel insights into the association between *PDIA4* and the development of CHD. The integration of clinical phenotyping, genetic analyses, and cellular functional evaluations suggests that PDIA4 may be involved in CHD pathogenic mechanisms. However, it should be noted that the current research has certain limitations. The GnomADv2_EAS database substantially reduces allelic frequency bias caused by population stratification, but it lacks detailed clinical and demographic covariates, which limits precise matching to disease cohorts. Moreover, although HUVECs are widely used as an *in vitro* surrogate for endothelial cells, they do not fully recapitulate the unique identities of endocardial cells or the complex intercellular crosstalk and organ-specific signaling microenvironment during cardiac development. In addition, the precise role of PDIA4 during cardiac morphogenesis requires further investigation in more representative models, such as heart organoids and *in vivo* functional studies.

## Data Availability

The datasets presented in this study can be found in online repositories. The names of the repository/repositories and accession number(s) can be found in the article/Supplementary Material.
