# Supplementary material for: Defects in PDIA4 increase individuals’ susceptibility to congenital heart disease
Source: Front Genet. 2026 Mar 4;17:1753969. doi: 10.3389/fgene.2026.1753969 (PMC12995189; doi:10.3389/fgene.2026.1753969)
Supplement: Supplementary file 1 [file DataSheet1.docx]

Supplementary Material

Defects in PDIA4 increase individuals’ susceptibility to congenital heart disease

**TableS1 Primer pairs for PDIA4 shRNA and primers used for RT-PCR analysis**

| **Primer name** | **Sequences 5’- 3’** |
| --- | --- |
| PDIA4-shRNA-F | GATCCGCCTGAGAAGATTACAAATTCTCGAGAATTTGTAATCTTCTCTCAGGTTTTTT |
| PDIA4-shRNA-R | AATTAAAAAACCTGAGAGAAGATTACAAATTCTCGAGAATTTGTAATCTTCTCTCAGGCG |
| RT-qPCR-PDIA4-F | GGCAGGCTGTAGACTACGAG |
| RT-qPCR -PDIA4-R | TTGGTCAACACAAGCGTGACT |
| RT-qPCR -GAPDH-F | GGAGCGAGATCCCTCCAAAAT |
| RT-qPCR -GAPDH-R | GGCTGTTGTCATACTTCTCATGG |
| RT-qPCR -DKK2-F | CTCACAGATCGGCAGTTCG |
| RT-qPCR-DKK2-R | ATGCCAGTCCTTGGTACATGC |
| RT-qPCR -CXXC4-F | ATGCACCACCGAAACGACTC |
| RT-qPCR -CXXC4-R | GCAGTGTTCAGGGGATAAGGT |
| RT-qPCR -SFRP1-F | ACGTGGGCTACAAGAAGATGG |
| RT-qPCR -SFRP1-R | CAGCGACACGGGTAGATGG |

**Table S2 Baseline Characteristics of 1792 patients with Congenital Heart Disease**

| Phenotypes | Septal defects and mild PS | Isolated abnormities of valves | Obstruction of right ventricular outfloe tract caused by abnormities of valves | Obstruction of left ventricular outflow tract | Conotruncal defects | Heterotaxy syndrome | Others |
| --- | --- | --- | --- | --- | --- | --- | --- |
| Case(number) | 643 | 59 | 111 | 68 | 693 | 25 | 193 |
| Age(Median, interquartile range) | 621（1-6097） | 2496.75（4-5735） | 2386.5（0-6040） | 2580.5（6-6075） | 705.75（1-6964） | 1930.25（277-6442） | 423.25（1-4967） |
| Male | 365 | 34 | 61 | 46 | 393 | 13 | 114 |
| Female | 278 | 25 | 50 | 22 | 300 | 12 | 79 |

**TableS3 Identification of 24 rare deleterious variants in gnomADv2_Exome_EAS database**

| Chromosome | Position | Reference | Alternate | RawScore | PHRED | Allele Count East Asian | Allele Number East Asian | MAF  (East asian) |
| --- | --- | --- | --- | --- | --- | --- | --- | --- |
| 7 | 148700891 | G | C | 3.225944 | 24.1 | 1 | 16632 | 6.01251E-05 |
| 7 | 148700983 | G | A | 4.282727 | 31 | 1 | 18350 | 5.44959E-05 |
| 7 | 148700996 | C | T | 2.539335 | 22.6 | 1 | 18318 | 5.45911E-05 |
| 7 | 148701031 | T | G | 3.449467 | 24.8 | 1 | 18380 | 5.4407E-05 |
| 7 | 148701047 | C | T | 2.445467 | 22.4 | 1 | 18384 | 5.43951E-05 |
| 7 | 148701107 | T | G | 3.029588 | 23.6 | 1 | 18394 | 5.43656E-05 |
| 7 | 148701137 | G | A | 3.383899 | 24.6 | 1 | 18394 | 5.43656E-05 |
| 7 | 148701236 | C | T | 3.451961 | 24.8 | 1 | 18394 | 5.43656E-05 |
| 7 | 148701255 | G | C | 3.051414 | 23.7 | 1 | 18386 | 5.43892E-05 |
| 7 | 148702293 | T | C | 3.104084 | 23.8 | 1 | 18394 | 5.43656E-05 |
| 7 | 148702344 | C | A | 4.420266 | 32 | 1 | 18394 | 5.43656E-05 |
| 7 | 148702445 | T | C | 3.120754 | 23.9 | 1 | 18394 | 5.43656E-05 |
| 7 | 148702449 | T | C | 2.661877 | 22.9 | 1 | 18394 | 5.43656E-05 |
| 7 | 148703079 | G | A | 3.755507 | 26 | 1 | 11122 | 8.99119E-05 |
| 7 | 148705298 | T | G | 3.324084 | 24.4 | 1 | 18388 | 5.43833E-05 |
| 7 | 148708938 | C | T | 4.236441 | 29.7 | 1 | 17340 | 5.76701E-05 |
| 7 | 148709085 | A | C | 3.942179 | 27.2 | 1 | 18096 | 5.52608E-05 |
| 7 | 148709277 | A | G | 3.181248 | 24 | 1 | 18394 | 5.43656E-05 |
| 7 | 148709390 | C | T | 2.672331 | 22.9 | 1 | 18394 | 5.43656E-05 |
| 7 | 148712126 | C | A | 2.326518 | 22.1 | 1 | 18392 | 5.43715E-05 |
| 7 | 148716101 | C | A | 4.152317 | 29.1 | 1 | 18392 | 5.43715E-05 |
| 7 | 148716152 | C | T | 4.069724 | 28.3 | 1 | 18394 | 5.43656E-05 |
| 7 | 148718141 | C | T | 4.024411 | 27.9 | 1 | 18392 | 5.43715E-05 |
| 7 | 148702428 | T | A | 8.050646 | 40 | 1 | 18394 | 5.43656E-05 |

**TableS4 Clinical information of CHD patients with** **rare pathogenic PDIA4 variants**

| Patient ID | Gender | Age(Days) | Clinical phenotype |
| --- | --- | --- | --- |
| 951 | M | 50 | VSD, ASD |
| 3759 | M | 20 | TAPVC，PDA |
| 1931 | M | 374 | Sling, TOF |
| 982 | F | 376 | VSD |
| 2207 | M | 171 | TOF |
| 2501 | M | 207 | TGA |
| 193 | M | 5 | TGA,VSD,PDA |
| B344 | M | 92 | TOF |
| B849 | F | 223 | TOF |
| B1038 | M | 312 | TGA |
| 1346 | M | 10 | TOF |
| B495 | F | 157 | TOF |
| 1154 | M | 171 | VSD |
| 243 | M | 192 | TOF |
| B108 | F | 9 | TOF |
| 2182 | M | 6 | TGA,PDA |
| 1143 | M | 552 | VSD |
| 0243 | M | 192 | TOF |
| B120 | M | 13 | TOF |

The following abbreviations were used: ASD, atrial septal defect; VSD,ventricular septal defect;PDA ,Patent Ductus Arteriosus;TAPVC, total anomalous pulmonary venous connection; TGA, transposition of the great arteries; TOF,tetralogy of fallot.

**TableS5** **Correlations between CHD Subtypes and rare pathogenic variants**

| CHD Subtype | Rare Pathogenic Variants, n (%) | Non-pathogenetic Variants, n | P Value | OR(95%CI) |
| --- | --- | --- | --- | --- |
| CTD | 14(1.01) | 1372 | 0.013 | 3.40(1.22,9.46) |
| Non CTD | 5(0.2) | 1667 |  |  |

The following abbreviations were used : CTD, Conotruncal defects; OR, odds ratio; CI, confidence interval
